# Effect of 10% fluoride on the remineralization of dentin *in situ*

**DOI:** 10.1590/1678-775720150239

**Published:** 2015

**Authors:** Mozhgan BIZHANG, Sabine KALETA-KRAGT, Preeti SINGH-HÜSGEN, Markus Jörg ALTENBURGER, Stefan ZIMMER

**Affiliations:** 1 - University Witten/Herdecke, Department of Operative and Preventive Dentistry, Witten, Germany.; 2 - Heinrich-Hein University Duesseldorf, Department of Operative and Preventive Dentistry and Periodontics, Duesseldorf, Germany.; 3 - Universitätsklinikum Freiburg, Department of Operative Dentistry and Periodontology, Freiburg, Germany.

**Keywords:** Microradiography, Tooth remineralization, Demineralization, In situ, Dentin, Fluorides, Chlorhexidine

## Abstract

**Objective:**

The purpose of this randomized, cross-over, *in situ* study was to determine the remineralization of demineralized dentin specimens after the application of a 10% fluoride (F-) or a 1% chlorhexidine–1% thymol (CHX–thymol) varnish.

**Material and Methods:**

Twelve individuals without current caries activity wore removable appliances in the lower jaw for a period of four weeks. Each appliance contained four human demineralized dentin specimens fixed on the buccal aspects. The dentin specimens were obtained from the cervical regions of extracted human third molars. After demineralization, half the surface of each specimen was covered with a nail varnish to serve as the reference surface. The dentin specimens were randomly assigned to one of the three groups: F-, CHX–thymol, and control (no treatment). Before the first treatment period and between the others, there were washout periods of one week. After each treatment phase, the changes in mineral content (vol% µm) and the lesion depths (µm) of the dentin slabs were determined by transverse microradiography (TMR). Data analysis was accomplished by the Kruskal-Wallis test and the Mann-Whitney U test (p<0.05).

**Results:**

The medians (25^th^/75^th^ percentile) of integrated mineral loss were 312.70 (203.0-628.7) for chlorhexidine varnish, 309.5 (109.8-665.8) for fluoride varnish, and -346.9 (-128.7 - -596.0) for the control group. The medians (25^th^/75^th^ percentile) of lesion depth were 13.6 (5.7-34.5) for chlorhexidine varnish, 16.5 (5.6-38.1) for fluoride varnish, and -14.2 (-4.5- -32.9) for the control group. Use of the 10% F- or 1% CHX–1% thymol varnishes resulted in significantly decreased mineral loss and lesion depth in dentin when compared with the control group. There were no statistically significant differences among the test groups.

**Conclusions:**

Within the limitations of this study, the results suggest that the effect of the treatment of demineralized dentin with 10% F- or 1% CHX–1% thymol is better than without any treatment.

## INTRODUCTION

Epidemiological studies have shown that root caries is common in adults^16^. Approximately 21.5% of adults between 35 and 44 years of age, and 45% of adults aged 65 to 74 who still have one or more of their own teeth also have decayed or filled root surfaces^16^. Oral prophylaxis and modern treatment strategies in conservative dentistry have led to an increase not only in the retention of healthy/sound teeth, but also in the number of teeth with recessions.This in turn has resulted in an increased risk of root caries^28^.

Fluoride plays an important role in the control of root caries by reducing the caries progression rate and by inducing the arrest of active lesions^29^. The effectiveness of fluoride in general shows a dose-response relation between fluoride concentration and the caries-preventive effect^3^. A review indicated that the application of high-concentration fluoride in the form of a dentifrice or mouthwash had a positive effect on root caries incidence^10^. A current systematic review of fluoride varnishes showed a substantial caries-inhibiting effect in both permanent and primary teeth^14^. However, the optimum fluoride concentration for the remineralization of root caries has not yet been defined^4^. Bifluoride is a commercially available high-concentration fluoride varnish used for topical fluoridation. It contains a combination of two fluorides: 5% sodium fluoride (NaF) (22,600 ppm F-) and 5% calcium fluoride (CaF2).

Non-fluoride agents may serve as adjunctive therapeutics for preventing, arresting, or even reversing dental caries. Studies have shown that CHX can be used for the control of plaque formation and caries prevention^15^. CHX has been reported to reduce the self-degradation of collagen fibrils by inhibiting host-derived protease activity in demineralized dentin. Theoretically, if the collagen fibril scaffold of demineralized dentin maintains its original cross-linkage pattern on treatment with CHX and appropriate supplementation with necessary mineral sources, dentin remineralization may occur in demineralized lesions^12^. One study showed that the application of the 0.2% and 2% CHX positively influences dentin remineralization^11^ ^,^ ^12^. One systematic review revealed that CHX may provide a beneficial effect in the prevention or treatment of root caries in patients with recessions without regular professional tooth cleaning and oral hygiene instructions. However, the strength of this recommendation is regarded as weak^23^. Investigations that compared 1% CHX–1% thymol varnish with topical fluoride applications have shown a similar effectiveness in dental caries prevention in the permanent teeth of teenagers^19^ ^,^ ^25^. In adults and older people, the application of a 1:1 mixture of 1% CHX–1% thymol varnish is said to reduce the incidence of root caries^21^ ^,^ ^26^ *. *CHX and fluoride have been found to have a significant effect on the remineralization of demineralized dentin under physiological conditions^11^. However, currently, to the best of our knowledge, there seems to be no study comparing the efficacy of a 1% CHX–1% thymol varnish with that of a high-concentration fluoride varnish on demineralized dentin. Therefore, the aim of this study was to evaluate the effect of 10% F^-^ and a 1% CHX–1% thymol varnish on the remineralization of demineralized dentin surfaces *in situ*, in comparison to that in a control group with no treatment. The null hypothesis to be tested was that there is no difference in the effectiveness of 10% F^-^ and 1% CHX–1% thymol in re-mineralization and in the formation of caries-like dentin lesions in comparison with a control group.

## MATERIAL AND METHODS

### Study population

Twelve volunteers (one male and eleven females) with a mean age of 32.5±11.8 years participated in this study. Fifteen participants were originally recruited, but only twelve were able to complete the study. The first 15 subjects, who were interested, agreed to participate in the study and fulfilled the inclusion and exclusion criteria, were required to sign the informed consent form and were enrolled in the study. The study was single-blinded, whereby the participants were blinded to their allocations. The subjects were all randomly divided into three groups. This study was a cross-over study; therefore, all 15 of the subjects have an equal chance of being assigned to the three groups. The specimens were analyzed by an examiner, who was also blinded.

All participants signed an informed consent form. No participant indicated the presence of systemic disease in his/her medical history. An intraoral examination confirmed that each participant had at least 22 natural teeth with no current caries activity, gingivitis, periodontal disease, or other oral pathology. None of the volunteers was using antibiotics or other medications that could have affected the salivary flow rate. The exclusion criteria were the presence of any systemic illness, pregnancy or breastfeeding, the use of fixed or removable orthodontic appliances, the use of a fluoride mouthrinse or professional fluoride application in the preceding two months, and hyposalivation. The salivary flow rate was quantitatively checked during oral examination, and a salivary flow rate under 0.1 mL/min was considered as hyposalivation. All participants had an unstimulated salivary flow rate of more than 0.1 mL/min. They were instructed to use the same toothpaste containing 1500 ppm Olaflur and Potassium Hydroxide (Elmex sensitiv; GABA, Lörrach, Germany) and toothbrush (Elmex Kariesschutz Inter X, GABA, Lörrach, Germany), starting four weeks prior to and continuing throughout the experimental period.

The study was approved by the ethical committee of the University of Düsseldorf, Germany (No. 3313), and the full trial protocol can be accessed there. The study has the following German Clinical Trials Register number: DRKS00005054. The sample size was calculated using G*Power (version 3.0; http://www.psycho.uni-duesseldorf.de/abteilungen/aap/ gpower3)^9^ prior to the commencement of this *in situ *study. In accordance with the results of a publication by Buchalla, et al.^6^ (2002), the number of participants was determined to be 15 (in anticipation of a 20% dropout rate) to determine a specimen quantity sufficient to achieve an adequate power of 80% and a defined significance level of 5% (p<0.05) for primary outcome.

### Specimens

We used 180 specimens from extracted caries-free human third molars for the intraoral remineralization model^24^. Dentin specimens were derived from the labial or lingual surface of the crown by a trephine bur with a 6 mm diameter. The enamel layer was entirely removed from this hard-tissue cylinder and validated by inspection of the specimens under a dissecting microscope (8x magnification). Since sterilization of specimens was essential for an ethical *in situ* study, the specimens were irradiated with a cobalt-60 source, at a total dose of 60 Gy^7^. After sterilization, all specimens were cleaned with a soft toothbrush under running tap water, and dried with air. We created artificial caries-like subsurface lesions by storing the specimens in a buffered demineralization solution (2.2 mN CaCl_2_.2H_2_O, 2.2 mM KH_2_PO_4_, 0.05 M acetic acid; pH adjusted to 4.5 with 10 M KOH)^24^ for five days. The demineralization process was carried out prior to the start of each phase. As the specimens were randomly assigned to one of the three groups, the demineralization of the specimens differed in each group. Half of each specimen surface was covered with an acid-resistant nail varnish (Jet-Set; Loreal, Karlsruhe, Germany) to serve as a reference surface.

(1) 1% CHX–1% thymol (n=60) (Cervitec plus^®^, 1% CHX-1% thymol; Ivoclar Vivadent, Schaan, Liechtenstein). The varnish contains ethanol, water, vinyl acetate copolymer and acrylate copolymer, thymol, and chlorhexidine diacetate.

After being cleaned and dried, the specimen surfaces were covered with Cervitec Plus by the Vivadent applicator. The varnish was dispersed with air for 30 s and left alone.

**Figure 1:**
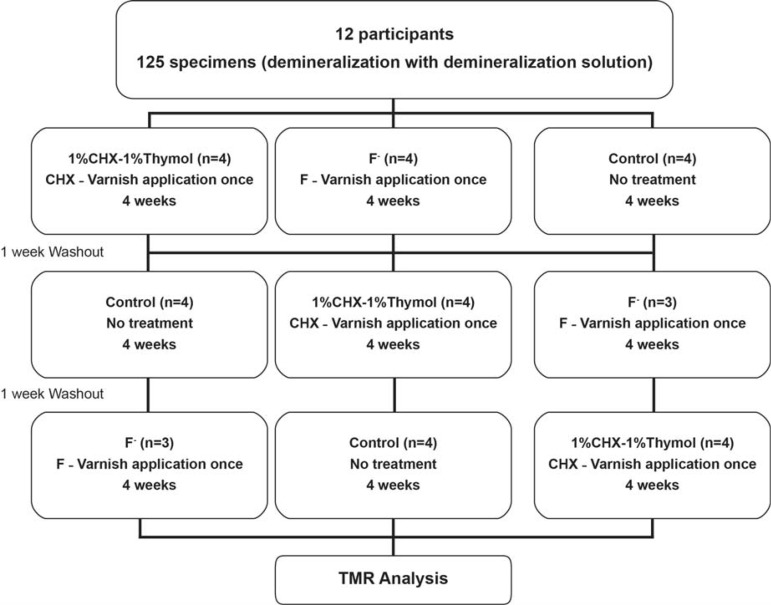
Study design

(2) 10% F^-^ (n=60) (Bifluoride 10^®^, VOCO GmbH, Cuxhaven, Germany). After being cleaned and dried, the specimen surfaces were covered with fluoride varnish. The varnish was left on specimen surfaces to absorb for 10–20 s, after which the surfaces were dried with air.

(3) Control group (n=60). The specimens were left untreated (control group) (Figure 1).

### *In situ* study

**Figure 2:**
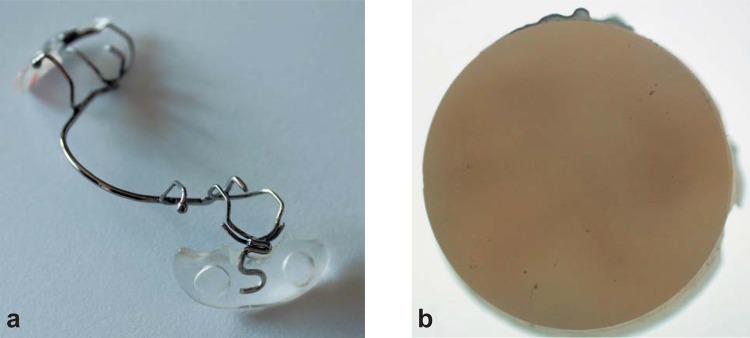
The intraoral appliance (a) and dentin specimen (b)

Each participant received an oral prophylaxis at the initial visit to establish a plaque- and calculus-free baseline. Thereafter, participants were randomly assigned to one of the three groups. A removable appliance was fabricated with an orthodontic wire (Dentaurum, Ispringen, Germany) and resin (Dentaurum) for the lower jaw of each participant. Four dentin specimens were inserted into both buccal aspects of the intraoral appliance, two specimens in the premolar and two in the molar region (Figure 2). The specimens of the appliances (not part of the experimental period) were stored in Ringer’s solution (0.9% NaCl Deltaselect GmbH, Pfullingen, Germany). The specimens were placed in the intraoral appliance with varnish coverage. After 24 h, the specimen surfaces were located 1 mm under the appliance surface to promote plaque accumulation. Four specimens were randomly chosen for every period and participant. Patients were instructed to wear the appliances approximately 24 h a day, to rinse their appliances under tap water, and to refrain from brushing the specimens. The appliance was meant to be removed only twice, during toothbrushing and during the three daily meals. At these times, the appliance was stored in a 10% sucrose solution at room temperature. This process was chosen to continue the demineralization process and avoid drying the specimens. The specimens were rinsed after the sucrose immersion. Before and between each four-week testing period, there was a 1-week washout period. Thus, after completion of all three groups, all participants should have worn the appliance for a total of 12 weeks.

At the end of each phase, all specimens were also examined for plaque build-up, which was found to be located on the upper surfaces. The presence of plaque was assessed by microscopy (8x magnification). A few specimens lost the varnish while wearing the appliances. The specimens with varnish were included only for the analysis. Therefore, the number of specimens in the three groups differed (Table 1).

**Table 1:** Median (25th/75th percentile) of Integrated mineral loss (IML, Vol%∙µm), lesion depth (µm), and change in IML and lesion depth of dentin treated with the two different varnishes and a control, after 4 weeks *in situ*. The negative value is a sign of demineralization. a, b Different letters within columns represented statistical differences (p<0.05)

Methods	IML (vol% x µm)	IML (vol% x µm)	Lesion depth (µm)	Lesion depth (µm)	Change in IML	Change in lesion depth
	(test area)	(reference area)	(test area)	(reference area)	(vol% x µm)	(µm)
10% F- (n=38) Median 25^th^/75^th^ percentile	744.9 298.58-1309.03	1259.9^a^ 788.7-2072.2	33.2^a^ 15.3-55.3	58.1^a^ 32.0-93.3	309.5^a^ 109.8-665.8	16.50^a^ 5.6-38.1
1% CHX-1% Thymol (n=43) Median 25^th^/75^th^ percentile	550.7^a^ 274.20-831.90	897.5^a^ 608.8-1436.6	27.3^a^ 10.4-33.0	36.6^a^ 22.1-59.3	312.7^a^ 203.0-628.7	13.6^a^ 5.7-34.5
Control (n=44) Median 25^th^/75^th ^percentile	1065.0^b^ 596.78-1547.10	584.2^b^ 357.3-969.0	42.0^b^ 26.3-70.6	26.90^b^ 18.7-39.4	346.9^b^ (-128.7)-(-596.0)	-14.2^b^ (-4.5)- (-32.9)

### Microradiography

Analysis of the specimens was performed by transverse microradiography (TMR), which provided direct measurements of the mineral content of the dental tissues examined^18^. TMR has been found to be more sensitive and valid than chemical analysis for the determination of mineral volume^18^. We prepared the dentin specimens for TMR by cleaning the exposed dentin surfaces with a soft toothbrush under running tap water to remove the plaque. Thereafter, the specimens were rinsed in deionized water for 2 min and sectioned perpendicular to the varnished area of the dentin. These transverse sections contained the experimental group and reference group in one section. Thin dentin sections (DS), 100 µm in thickness, 1.5–2.0 mm in width, and 5 mm in length, were obtained and polished with sandpaper (4000 grit). The DS samples were microradiographed together with an aluminum reference step-wedge on a high-speed holographic film (SO-253: Kodak, Stuttgart, Germany). The x-ray source (PW 1830/40; Philips, Kassel, Germany) had a nickel-filtered copper (CuKα) radiation , and the x-ray settings were 20 kV and 20 mA for 12 s. The distance between the radiation source and the film was fixed at 34 cm.

Microradiographs were scanned with a digital image analyzing system (CCD Video camera Modul XC77E; Sony, Japan) connected to a universal microscope (Axioplan; Zeiss, Oberkochen, Germany) and a personal computer. After calibration of the image, integrated mineral loss (∆Z; vol%∙µm) and lesion depth (µm) were measured with the recommended software (TMR for Windows, Release 1.24e; Inspektor Research Systems, Amsterdam, The Netherlands) with predefined settings as described in an earlier publication^4^. Mineral gain was calculated as the difference in mineral loss between the exposed and reference demineralized areas. Similarly, the lesion depth reduction was calculated between the exposed and reference areas in the center of each area, which was 1.25 mm away from the perimeter of the dentin disc, thus resulting in sites of analysis that were 2.5 mm apart. Positive values were interpreted as remineralization, and negative values as demineralization.

At the end of the 4-week *in situ* period, the specimens were removed from the appliance and stored in Ringer’s solution (0.9% NaCl, Deltaselect GmbH, Pfullingen, Germany) before being analyzed, but were kept dry during measurements.

### Statistical methods

All statistical analyses were performed with SPSS, version 18.0 for Windows. The distribution of the data (mean and standard deviation) for each group was analyzed by the Kolmogorov-Smirnov test. Data comparison among the three groups regarding integrated mineral loss and lesion depth was accomplished by the Kruskal-Wallis and Mann-Whitney U tests. The comparison revealed that the data passed the normality test, but the variances were not homogeneous. Therefore, these data were compared by the Kruskal-Wallis test followed by Dunn’s multiple-comparison test (since the specimens were selected from third molars of different participants). The tests were performed with α=0.05 to test for significant differences among the three groups.

## RESULTS

The data on lesion depths and mineral gain were not normally distributed. The dropout rate was 20%, with three individuals discontinuing the study. The reasons for dropout were illness (one participant) and non-acceptance of the removable appliance (two participants). One participant inserted the appliance only for 1% CHX–1% thymol, and the control specimens and received orthodontic treatment after the second period. A few specimens were lost. Thus, only 38 specimens were analyzed for the F^- ^group, 43 for the 1% CHX–1% thymol group, and 44 for the control group (Table 1). All artificially demineralized specimens showed homogeneous subsurface lesions. The medians (25^th^/75^th^ percentile) of integrated mineral loss were 312.70 (203.0-628.7) for chlorhexidine varnish, 309.5 (109.8-665.8) for fluoride varnish, and -346.9 (-128.7 - -596.0) for the control group. The medians (25^th^/75^th^ percentile) of lesion depth were 13.6 (5.7-34.5) for chlorhexidine varnish, 16.5 (5.6-38.1) for fluoride varnish, and -14.2 (-4.5- -32.9) for the control group. Use of the 10% F^- ^ or 1% CHX–1% thymol varnishes resulted in significantly decreased mineral loss and lesion depth in dentin compared with the control group.

**Figure 3:**
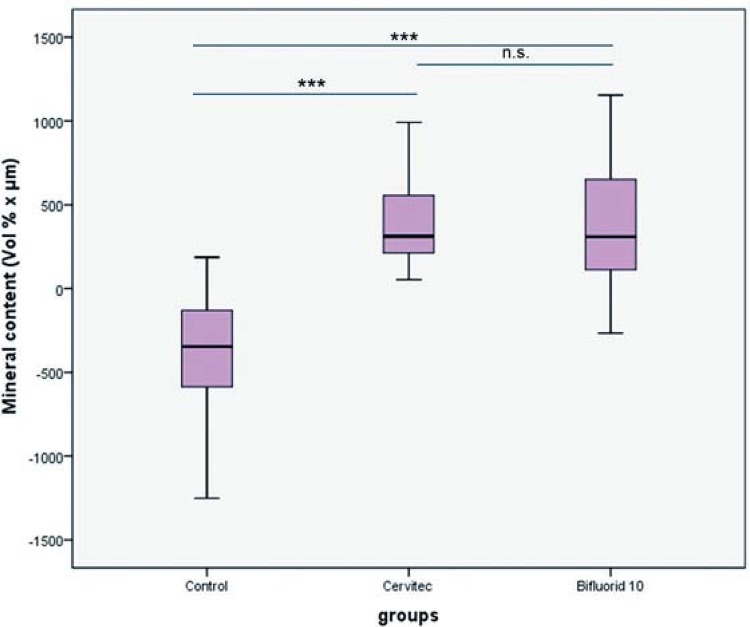
Median of the mineral content (Vol%∙µm) in a thin section of the dentin specimens *in situ* (***p<0.001; n.s.: no statistically significant differences)

**Figure 4:**
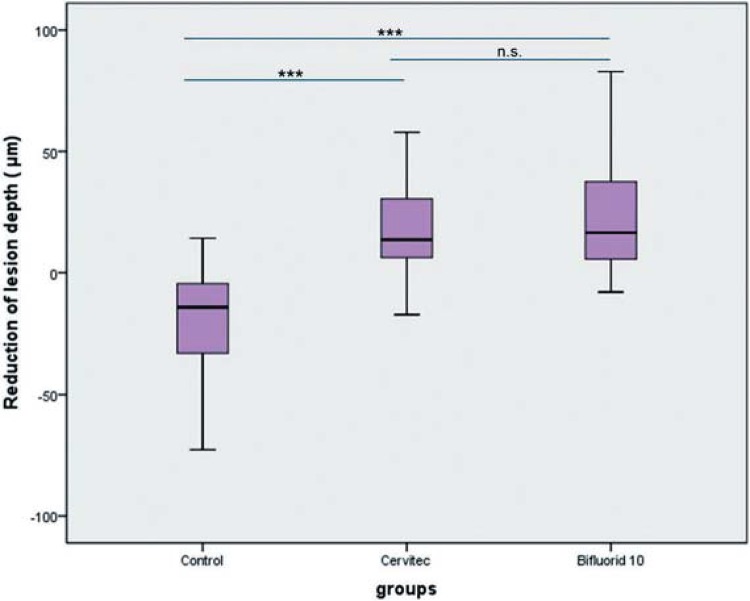
Median of the reduction in lesion depth (µm) in a thin section of the dentin specimens *in situ* (***p<0.001; n.s.: no statistically significant differences)

**Figure 5:**
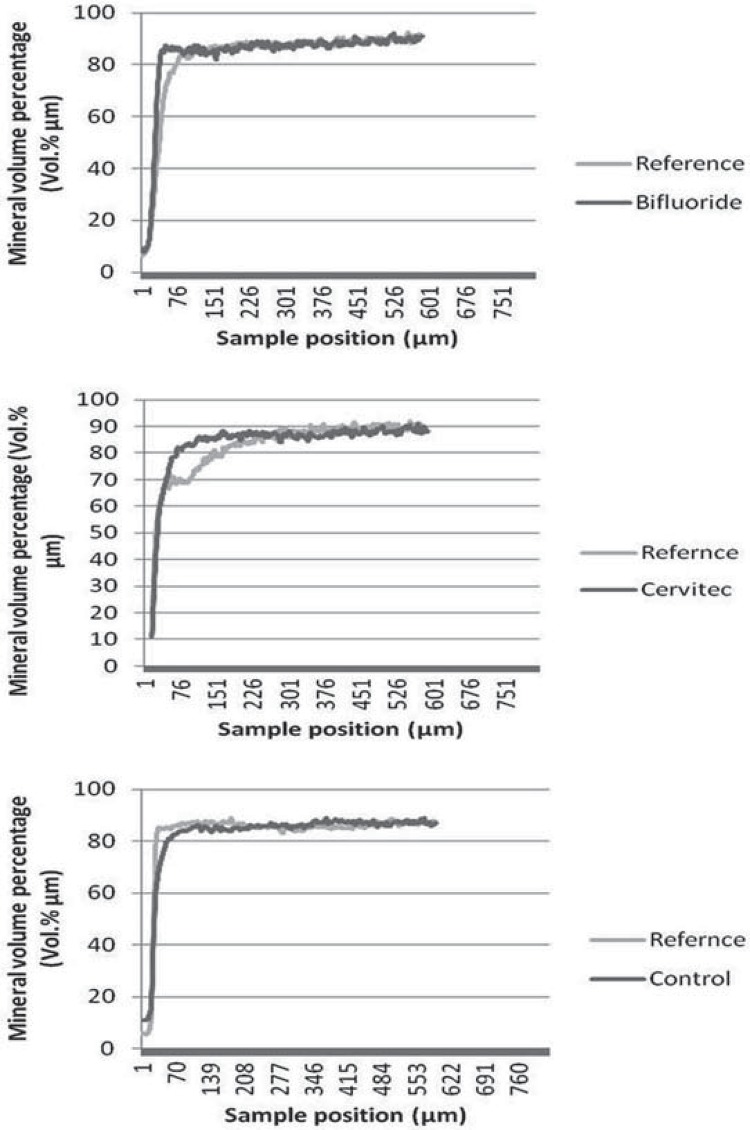
Profile examples of the mineral volume percentage

The changes in integrated mineral loss and in lesion depth were significantly higher in the 10% F^- ^ and 1% CHX–1% thymol groups compared with those in the control group (Table 1, Figures 3 and 4). Figure 5 shows three samples of mineral profiles of lesions remineralized with either 1% CHX–1% thymol or with 10% F^- ^or in the control group. However, no statistically significant differences were found between the 10% F^- ^ and 1% CHX–1% thymol for changes in integrated mineral loss and in lesion depth. Nonetheless, a single application of 10% F^- ^ and 1% CHX–1% thymol to initial dentin lesions increased the reduction in lesion depth and decreased the mineral loss. No adverse events were observed in this study.

## DISCUSSION

This study showed that a single application of 10% F^- ^ or 1% CHX–1% thymol treatment was more effective in promoting the remineralization of dentin than the no-treatment control group. Hence, the primary outcome of this study was confirmed. The results of the fluoride group were comparable to the known results of the effectivity of topical application of highly concentrated fluoride formulations in caries prevention^14^. The caries-preventive effect of fluoride is mainly attributed to its effects on demineralization/remineralization at the tooth–oral fluid interface^1^. The primary mode of fluoride action is the inhibition of demineralization and enhancement of remineralization^22^. *In vitro* and *in vivo* enamel or dentin remineralization experiments have demonstrated that, with increasing concentrations of fluoride, the calcium loss from enamel and dentin was reversed under acidic conditions, thus promoting remineralization and inhibiting demineralization^2^ ^,^ ^4^ ^,^ ^8^. The results of the present study with a higher fluoride concentration, a combination of 5% NaF (equal to 22,600 ppm fluoride) and 5% CaF_2, _were congruent with these studies.

The CHX mechanism may assert that the interaction between CHX and apatit is not adsorptive, but reactive. CHX may be due to electrostatic links with the phosphate groups in the hydroxyapatite of the dentin and saliva, which could favor the precipitation of phosphate salts on the reactive surface of demineralized dentin^17^ ^,^ ^20^. An *in vitro* study reported the direct effects of CHX on the remineralization of demineralized dentin. It showed that the application of 0.2% and 2% CHX, which inhibits the matrix metalloproteinase (MMP) activity, seemed to be effective in promoting the remineralization of demineralized dentin. CHX could keep the collagen cross-linkage sound by inhibiting the MMP activity. Remineralization would then happen around the remaining mineral crystals by obtaining mineral sources from a simulated body fluid (SBF)^11^. 1% CHX–1% thymol has inhibitory effects on the demineralization of dentin^5^. Our results also confirm that 1% CHX–1% thymol was effective in the remineralization of dentin compared with the control group. Current studies have reported that the artificial protein formed due to the mineralization process on human dentin provides a general strategy to prepare various promising restorative materials for biomineralized hard tissues such as bone and teeth. These agents could be potentially applicable in a clinical situation in the future, to remineralize the demineralized dentin^13^ ^,^ ^30^.

Generally, our results are in agreement with the other studies, but have some limitations regarding the clinical situation. Caries is a complex phenomenon influenced by systemic defense factors and a number of external ones. There is no doubt that these factors differ in every individual and play a significant role in dentin demineralization. Classical risk factors of caries, such as eating habits, salivary flow rate, and oral hygiene, are individual-dependent. Therefore, 12 individuals were chosen for this study. In addition, root caries is a dental disease caused by exposure of the root surfaces, thus allowing the microorganisms to erode the dentin directly. The structure and composition of root-dentin and crown-dentin is not similar. Older individuals show a decrease in the components and quality of saliva. The participants of this study were young and did not present root caries, but they carried demineralized dentin specimens, as in another study^27^. Furthermore, the dentin used in the study was crown-dentin (below enamel). Both the aforementioned factors were limitations of this study. Another limitation was that the subjects were instructed to remove the appliance only during tooth brushing (twice a day) and meals (four times a day for a maximum of 1 h each). The standardized 10% sucrose solution was renewed every day.

Before and between each four-week testing period, there was a 1-week washout period to avoid a sequence effect. This is a common method of minimizing carry-over effects, and ensures that the participants start each test period under the same conditions.

In our study, the mineral loss after demineralization in the control group was more than that of the chlorhexidine and fluoride groups. It was decided to randomly distribute the specimens before the start of each phase, as extracted human teeth usually exhibit an inconsistent age and source; these factors may bring about a variance in the tooth composition, which then leads to larger variations in the demineralization process. Therefore, the remineralization progression was not linear during the whole treatment, which may have led to a different effect in different stages with the same intervention.

## CONCLUSION

Based on the results and within the limitations of this study, we conclude that the single use of 10% F^- ^or 1% CHX–1% thymol have a considerably better effect on the remineralization of demineralized dentin surfaces *in situ *than a no-treatment control group. These may be beneficial in preventing dentin demineralization. Since this preliminary investigation may provide a valuable insight into the clinical indication of a high-concentration fluoride varnish, future research in the form of clinical trials would be valuable. Subsequent studies dedicated to analyzing the frequency of applications and the investigation of the best method to exert a prolonged anti-caries effect are suggested.
